# Comprehensive mapping of cognitive and emotion networks in stress, anxiety, and depression implicates the precuneus as a critical hub

**DOI:** 10.21203/rs.3.rs-7200801/v1

**Published:** 2025-08-26

**Authors:** Mohammad Sendi, Mark Halko, Jenna Traynor, Joshua Brown, Marc Copersino, Lisa Nickerson, Nathaniel Harnett, Sanne van Rooij, Diego Pizzagalli, Stacey House, Francesca Beaudoin, Xinming An, Thomas Neylan, Gari Clifford, Tanja Jovanovic, Sarah Linnstaedt, Laura Germine, Kenneth Bollen, Scott Rauch, John Haran, Alan Storrow, Christopher Lewandowski, Paul Musey, Phyllis Hendry, Sophia Sheikh, Christopher Jones, Brittany Punches, Robert Swor, Nina Gentile, Lauren Hudak, Jose Pascual, Mark Seamon, Erica Harris, Claire Pearson, David Peak, Robert Domeier, Niels Rathlev, Brian O’Neil, Paulina Sergot, Leon Sanchez, Steven Bruce, John Sheridan, Steven Harte, Ronald Kessler, Karestan Koenen, Elizabeth Phelps, David Salat, Helen Mayberg, Samuel McLean, Jennifer Stevens, Vince Calhoun, Kerry Ressler, Daniel Dillon

**Affiliations:** Harvard Medical School/McLean Hospital; Harvard Medical School/McLean Hospital; Harvard Medical School/McLean Hospital; Harvard Medical School/McLean Hospital; Harvard Medical School/McLean Hospital; Mass General Brigham; McLean Hospital; Emory University; University of California; Washington University School of Medicine; The Alpert Medical School of Brown University, Rhode Island Hospital and The Miriam Hospital; University of North Carolina at Chapel Hill; San Francisco VA Healthcare System; University of California San Francisco; Emory University School of Medicine; Georgia Institute of Technology; Wayne State University; University of North Carolina at Chapel Hill; McLean Hospital; University of North Carolina Chapel Hill; McLean Hospital; University of Massachusetts Medical School; Vanderbilt University Medical Center; Henry Ford Health; Indiana University School of Medicine; University of Florida College of Medicine -Jacksonville; University of Florida College of Medicine -Jacksonville; Cooper Medical School of Rowan University; University of Cincinnati College of Medicine & University of Cincinnati College of Nursing; Beaumont Hospital; Lewis Katz School of Medicine at Temple University; Emory University School of Medicine; Perelman School of Medicine at the University of Pennsylvania; University of Pennsylvania; Einstein Medical Center; Wayne State University; Ascension St. John Hospital; Massachusetts General Hospital; Saint Joseph Mercy Hospital; University of Massachusetts Medical School-Baystate; Wayne State University School of Medicine; McGovern Medical School at UTHealth; Beth Israel Deaconess Medical Center; Harvard Medical School; University of Missouri-St. Louis; The Ohio State University; University of Michigan; Harvard Medical School; Broad Institute of MIT and Harvard; Harvard University; Harvard Medical School; Icahn School of Medicine at Mount Sinai; University of North Carolina at Chapel Hill; Emory University; McLean Hospital; McLean Hospital/Harvard Medical School

## Abstract

Stress-related conditions disrupt cognition and emotion regulation and can result in psychiatric illness, but the neural circuit-level changes that can explain these broad effects remain unclear. To address this issue, we paired population-grounded discovery with out-of-sample testing. Using resting-state fMRI from > 14,000 healthy adults in the UK Biobank, we derived connectivity profiles tied to cognition (reaction time, numeric memory), and proxies of emotion dysregulation (neuroticism, anhedonia). We then applied the profiles to a trauma-exposed cohort (N = 306) to assess symptom relevance. Associations with stress, anxiety, and depression concentrated in a subset of circuit motifs, two of which recurred: (1) hyperintegration between the default mode and control/limbic interfaces, as well as (2) hypointegration between the default mode and visual interfaces, both tracked higher symptom burden. Static and dynamic analyses converged on the precuneus as a critical hub: stronger precuneus–visual coupling and greater occupancy of a precuneus-engaged dynamic state were related to lower symptoms, whereas the opposing state was related to higher burden. This novel hybrid approach—discover-thenproject—thus yielded interpretable markers of circuit dysfunction that generalized to post-trauma psychopathology. Furthermore, the approach identified the precuneus as a potential target for mechanistically informed interventions.

## Introduction

Anxiety, depression, and post-traumatic stress disorder (PTSD) are all characterized by challenges with cognition and emotion regulation, including difficulties sustaining attention^1^, holding and updating information in working memory^2^, shifting between goals^3^, and managing distress^4–6^. These failures rarely occur in isolation^7–9^. Moment-to-moment behavior depends on coordinated interactions among large-scale neural systems supporting control, internal mentation, and sensorimotor integration^10–12^, and what appears as a “cognitive lapse” may actually reflglect inability to regulate distressing emotions; similarly, what seems to be emotional volatility may partly reflglect cognitive overload. In the worst cases, negative thoughts consume limited control resources and maladaptive policies are reinforced, establishing feedback loops that can elicit symptoms of psychiatric illness and may lead to functional decline^13–16^. Although transdiagnostic frameworks increasingly recognize this complexity, neural accounts that address cognition and emotion simultaneously remain rare^17–20^.

Several neural circuits influence both cognition and emotion^21–26^. For example, while the cognitive control (CC) network—involving key regions such as the dorsolateral prefrontal cortex and inferior parietal lobule—underpins attentional focus and working memory^27,28^, it is also critical for emotion regulation^29,30^. Similarly, the default mode (DM) network, which is implicated in depressive rumination^31,32^, is also well-known for its role in cognition^33^ and cognitive transitions^34^. The DM likely contributes to emotion dysregulation via its role in repetitive negative thinking, or rumination^35^, but it is also integral to complex cognitive tasks such as planning, decision-making, and memory retrieval^33^. Another example is the sensorimotor (SM) network. The SM network is traditionally known for movement and sensory processing, but it also contributes to cognition and emotion. Recent studies link SM connectivity to episodic memory in older adults^36^ and higher-order functions such as selective attention^37,38^ and decision-making^39,40^. It also supports emotion regulation by processing bodily sensations tied to emotional states^41–43^. Dysfunction in this network is linked to emotion regulation deficits in psychiatric disorders^44^, including depression^45^ and PTSD^46,47^, and increased impulsivity in PTSD and related conditions^48^, underscoring its importance in mental health. Together, these findings highlight the complex interplay of cognition and emotion networks, underscoring the need for comprehensive mapping to understand the neural basis of stress, anxiety, and depression.

Two limitations in the literature are persistent. First, most studies interrogate cognition and emotion regulation separately, often in modest or clinically homogeneous samples, yielding findings that are difficult to integrate across tasks, measures, and diagnoses^49–51^. Second, polarization between hypothesis-driven analyses (high interpretability, low discovery)^52,53^ and fully data-driven discovery (high discovery, limited mechanistic mapping)^54,55^ impedes progress. As a result, it remains unclear how brain networks including CC, DM, visual (VIS), and SM co-configure in the healthy population^56–58^ to support adaptive cognition–emotion and, critically, along which axes trauma-exposed individuals deviate as symptoms emerge^59,60^.

Here we address these gaps with a hybrid strategy. We leverage resting-state fMRI (rs-fMRI) from > 14,000 healthy adults in the UK Biobank (UKBB)^61^ to derive robust functional connectivity profiles linked to cognition (choice reaction time; numeric memory) and to emotion (dys)regulation proxies (neuroticism; anhedonia). These profiles yield a normative reference for brain coordination anchored to behavioral variation at scale. We then project an independent cohort of recently trauma-exposed civilians from Advancing Understanding of RecOvery afteR traumA or AURORA^62^ (N = 306) into this reference space, to test whether early symptom burden links with changes in these circuits, and with their temporal dynamics (dFNC), two weeks after trauma.

This approach advances prior work in four ways: (1) Scale and generalizability: by anchoring circuit axes to behavioral variance in > 14k healthy adults and testing them in an external trauma cohort, we move beyond single-site, single-diagnosis reports toward population-relevant, out-of-sample generalization; (2) Interpretability with discovery: by first discovering functional connectivity and behavior associations in the reference population and then constraining tests in AURORA to those circuits, we preserve interpretability while retaining sensitivity to unanticipated relationships; (3) Dynamics: by quantifying state occupancy of cognition- and emotion regulation-linked networks, we identify dynamic signatures with opposing clinical valence that static FC alone cannot reveal; and (4) Mechanistic leverage: by converging on precuneus-centered functional coupling as a critical node, we identify a target for neurotherapeutic strategies. Together, these strengths highlight the value of integrating population mapping of cognition–emotion coupling to early post-trauma symptom dimensions, as a means to identify targets for intervention.

## Results

### Study population

**Figure 1:**
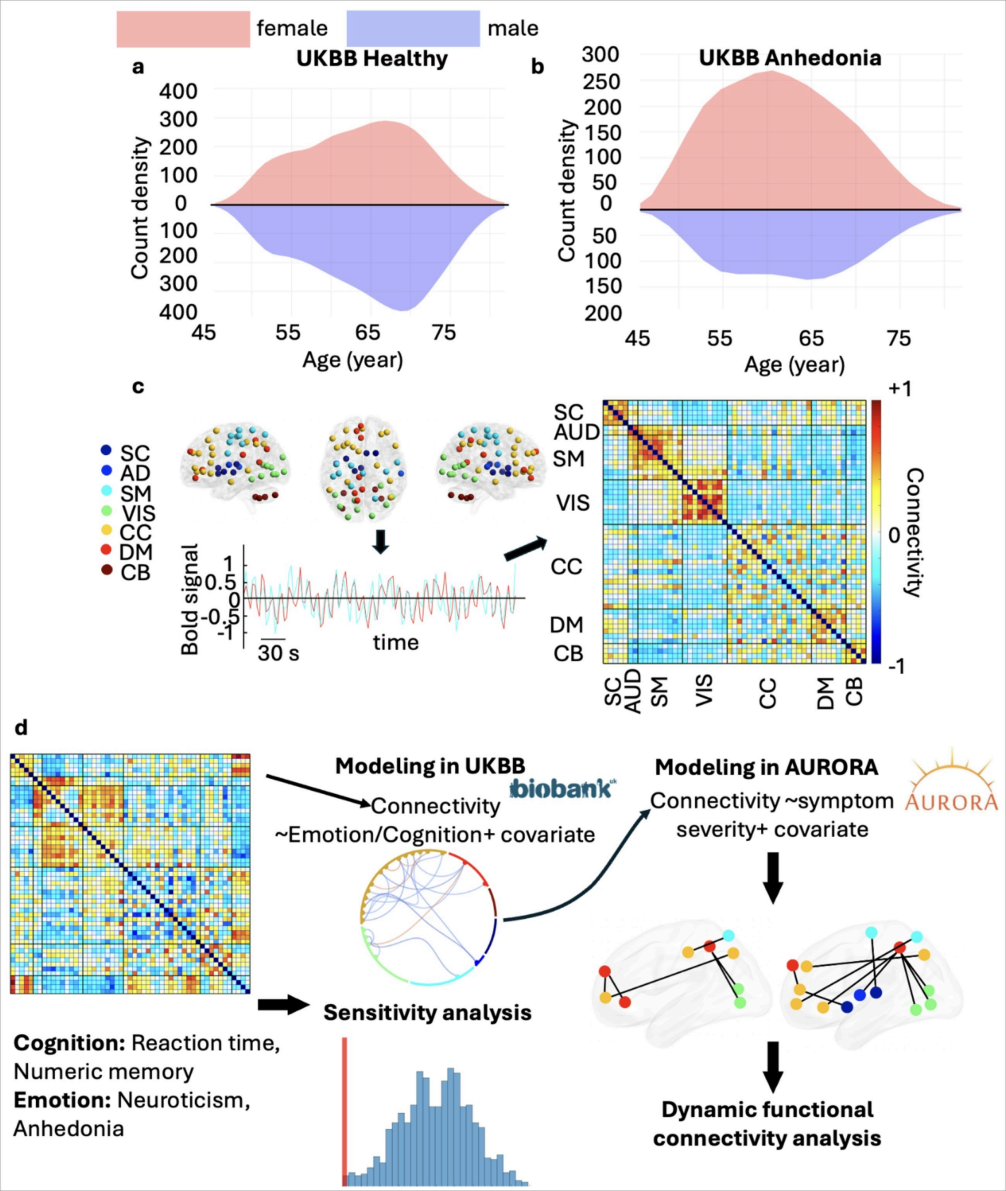
Overall study and analytic design. **a)** UKBB age count density for healthy individuals. **b)** UKBB age count density for individuals with anhedonia, with pink representing females and blue representing males. **c)** Functional connectivity between pairs of regions was calculated using the NeuroMark template, resulting in a 53×53 matrix or 1,378 functional connectivity measures. A representative functional connectivity map averaged across UKBB participants was estimated using the NeuroMark pipeline. SC: subcortical network, AUD: auditory network, SM: sensorimotor network, VIS: visual sensory network, CC: cognitive control network, DM: default mode network, CB: cerebellar network. **d)** General linear models (GLMs) were developed to examine relationships between 1,378 functional connectivity measures with cognitive measures (reaction time and working memory) and emotion measures (neuroticism and anhedonia) from UKBB, controlling for age, age^2^, sex, age × sex interaction, and collection site. We identified functional connectivities with significant links to cognitive and emotion measures after FDR correction. Additional GLMs were used to explore associations between the brain networks identified using UKBB data and the severity of stress, anxiety, and depression symptoms in trauma-exposed individuals from the AURORA data. Functional connectivities with significant links to symptom severity measures were identified after FDR correction.

We used data from the UKBB^61^ and AURORA^62^ studies, with UKBB serving as the reference cohort for model development and AURORA testing its generalizability in trauma-exposed individuals. In UKBB, we included healthy adults 45–85 year old who met MRI quality standards and excluded participants with ICD-10 diagnoses of psychiatric or neurological conditions, substance use disorders, or those who had received treatment for anxiety or depression (see *Supplementary Materials*). The final sample included 14,047 individuals (6,362 females; mean age = 64 ± 8 years; **Supplementary Fig. 1**). We selected two UKBB measures, choice reaction time and numeric memory, as indicators of cognitive speed and capacity^63^. We used neuroticism and anhedonia as proxies for emotion *dys*regulation: neuroticism captures a tendency toward negative emotions, while anhedonia reflects diminished interest or pleasure and is a core symptom of depression. Figure 1a shows the age distribution, and **Supplementary Table 1** summarizes demographic and behavioral data.

Our second dataset came from the longitudinal AURORA study, which recruited 2,943 civilians with recent trauma-exposure from one of 29 participating Emergency Departments across the United States. Participants provided clinical data at intervals of 2 weeks (WK2), 4 weeks (WK4), 3 months (M3), 6 months (M6), and 12 months (M12), relative to their traumatic event, and many reported symptoms of stress, depression, and anxiety. Stress was assessed with the PTSD Checklist for DSM-5 (PCL-5)^64^, whereas anxiety and depression were evaluated using the PROMIS Anxiety and PROMIS Depression scales^65^. Resting-state fMRI data were collected from approximately 400 participants at WK2. Participants with low-quality resting-state fMRI or missing clinical information at the time of imaging were excluded, resulting in 306 participants (198 females) being included in this analysis, with a mean age of 34±13 years (see **Supplementary Fig. 2** and **Supplementary Table 2**).

### Brain networks linked to cognitive function in the healthy UKBB sample

By applying group-ICA to resting state fMRI data, we first identified 53 brain regions (**Supplementary Table 3**) for each participant and grouped them into seven resting-state networks: the subcortical (SC), auditory (AUD), sensorimotor (SM), visual sensory (VIS), cognitive control (CC), default mode (DM), and cerebellar (CB) networks (**Supplementary Fig. 3**). After calculating functional connectivity among these 53 networks, we obtained 1,378 measures per participant (Fig. 1c). We then developed general linear models (GLMs) to examine relationships between two cognitive measures, choice reaction time (reaction time; **Supplementary Fig. 4a**) and numeric memory (**Supplementary Fig. 4b**), while including age, age^2^, sex, the age × sex interaction, and the site of data collection as covariates (Fig. 1d).

**Figure 2:**
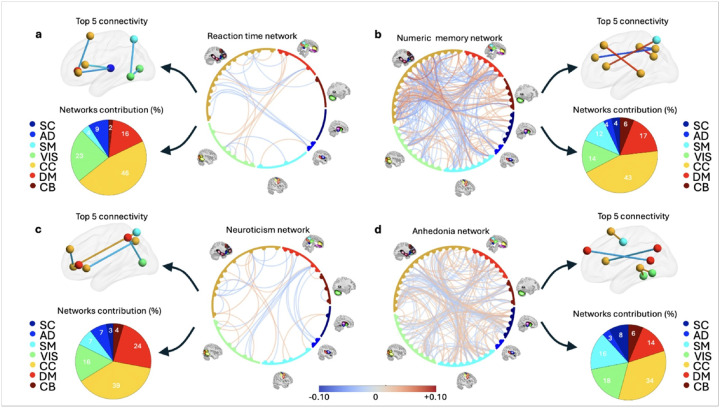
Brain networks associated with cognition and emotion in healthy individuals from UKBB. **a)** Visualization of brain networks linked to variations in mean (correct) choice reaction time. Red lines indicate longer reaction times (suggesting poorer cognitive performance), while blue lines represent shorter reaction times (suggesting better cognitive performance). The CC, SM, VIS, and DM networks are highlighted, with a focus on CC’s prominent role. **b)**Brain networks involved in predicting numeric memory performance. In this analysis, we used the reverse of maximum remembered digits. Then, red lines are associated with lower scores and indicate worse cognitive performance, whereas blue lines denote higher scores and indicate better cognitive performance. **c)** This panel shows the brain networks predicting neuroticism, used as a proxy for emotion regulation challenges, in the UKBB sample. Red lines indicate higher neuroticism, associated with greater emotion regulation challenges, while blue lines represent lower neuroticism, indicative of fewer emotion regulation challenges. Notable contributions from the DM, particularly the precuneus, as well as the CC and VIS, are consistent with an interplay between self-referential processing and cognitive control being critical to the expression of neurotic traits. **d)** This panel illustrates the brain networks involved in predicting anhedonia, with red lines denoting higher connectivity (linked to increased emotion regulation challenges) and blue lines showing lower connectivity (associated with fewer emotion regulation challenges). The involvement of DM, CC, VIS, and SM underscores their combined impact on anhedonia, providing insights into potential therapeutic targets to enhance emotion regulation and cognitive processing in individuals experiencing these symptoms.

Figure 2a displays 28 brain connections that remained significant after FDR correction in the model linking reaction time to functional connectivity in UKBB (**Supplementary Data 1**). In this figure, red lines indicate connections associated with longer reaction times, suggesting reduced cognitive function, while blue lines indicate connections associated with shorter reaction times, indicative of enhanced cognitive function. Additionally, the top five connectivity measures showing the strongest association with reaction time are displayed, along with the percentage contribution of each network in the model. Notably, the findings indicate the CC network is the most influential, accounting for 46% of all connections related to reaction time. Also, three of the top five connectivity measures originate from this network. Numerous connections between the CC network and other networks, including the VIS, SM, and DM, are also shown. Specifically, increased connectivity between SM and VIS, and between the DM and CC, were associated with reduced reaction time, suggesting enhanced cognitive function. To confirm the robustness of the associations between the 28 connectivity variables and reaction time, we performed 1,000 permutations by randomly shuffling reaction time values and rerunning the regression. All 28 variables remained significant (empirical p ≤ 0.05), suggesting the findings are likely not due to chance and may reflect meaningful neural markers of cognitive function.

Figure 2b shows brain networks associated with numeric memory scores after FDR correction. From 1,378 connectivity measures, 239 were identified as relevant to numeric memory (**Supplementary Data 2**). Red lines indicate connections linked to poorer performance, while blue lines reflect associations with better numeric memory, suggesting enhanced cognitive function. Notably, approximately 43% of these connections involved the CC, either within the CC network or between the CC network and other networks. The five most significant associations all originated from the CC and include: a connection between right inferior parietal lobule and left inferior parietal lobule; a connection between right inferior parietal lobule and middle frontal gyrus; a connection between right superior parietal lobule and right inferior frontal gyrus; a connection between left superior frontal gyrus and left hippocampus; and a connection between right inferior parietal lobule and right inferior parietal lobule. To confirm reliability, we ran a permutation test by shuffling memory scores 1,000 times and comparing p-values. All 239 functional connectivity variables remained significantly linked to memory (*p* ≤ 0.05), supporting their role as neural markers of cognitive performance.

### Brain networks linked to emotion dysregulation in the healthy UKBB sample

We developed multiple GLMs to investigate relationships between our chosen indices of emotion dysregulation, focusing on neuroticism (**Supplementary Fig. 4c**) and anhedonia. Our model again included age, age^2^, sex, the age × sex interaction, and the site of data collection as covariates. Figure 2c illustrates 34 functional connectivity links with neuroticism that remained significant after FDR correction (**Supplementary Data 3**). Red lines are connections linked to higher neuroticism, while blue lines are associated with lower neuroticism. The top five functional connectivity measures include: a connection between superior medial frontal gyrus and right inferior frontal gyrus; a connection between right inferior parietal lobule and left insula; a connection between right superior parietal lobule and right inferior frontal gyrus; connectivity between right middle occipital gyrus and precuneus; and connectivity between right inferior frontal gyrus and left anterior cingulate cortex. Notably, nine of 34 connections between the DM network and the rest of the brain involve the precuneus. All 34 functional connectivity variables remained significantly associated with neuroticism across 1,000 permutations (p ≤ 0.05), supporting their relevance as potential neural markers of emotion dysregulation.

Because a dimensional measure of anhedonia was not available, in the UKBB dataset we identified N = 4,379 participants with a positive (i.e., present versus absent) anhedonia score (see Methods and *Supplementary Materials*). To explore brain networks associated with anhedonia, we compared these participants with a healthy control group from the UKBB. Figure 2d illustrates all 262 (out of 1,378) connectivity links with anhedonia that remained significant after applying FDR correction (**Supplementary Data 4**). Red lines indicate connections linked to the presence of anhedonia, while blue lines are connections linked to its absence. As shown, the CC accounted for 34% of the connections, while the VIS, SM, and DM networks contributed 18%, 16%, and 14%, respectively. The top five brain functional connections linked to anhedonia include: a connection between right fusiform gyrus and left hippocampus; a connection between the precuneus and posterior cingulate cortex; a connection between left middle temporal gyrus and left hippocampus; a connection between right inferior frontal gyrus and precuneus; and a connection between left precentral gyrus and left supplementary motor area. All 262 connectivity variables remained significantly associated with anhedonia (*p* ≤ 0.05) after 1,000 permutations.

#### Networks linked to cognitive function in healthy adults are associated with symptom severity in trauma-exposed individuals

**Figure 3:**
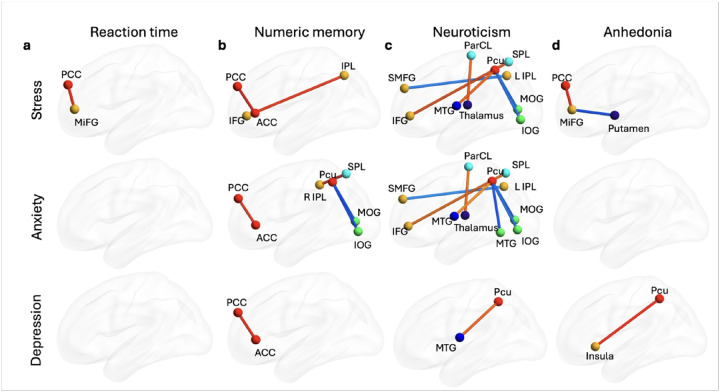
Brain networks associated with cognition and emotion in AURORA. **a) Links between reaction time networks and symptom severity in AURORA**. Of the 28 UKBB connectivity measures linked to processing speed, only connectivity between left posterior cingulate cortex and left middle frontal gyrus was significantly associated with stress in AURORA after FDR correction (β = 23.73, SE = 6.20, r = 0.21, FDR p = 0.0047). **b) Link between numeric memory networks and symptom severity in AURORA**. Connectivity between left posterior cingulate cortex and left anterior cingulate cortex was significantly associated with stress (β = 24.03, SE = 5.54, r = 0.20, FDR p = 3e-4), anxiety (β = 5.00, SE = 1.37, r = 0.20, FDR p = 0.030), and depression (β = 11.81, SE = 3.08, r = 0.21, FDR p = 0.038) in AURORA. Additionally, right inferior parietal lobe/right inferior frontal gyrus connectivity was linked to stress (β = 23.18, SE = 6.33, r = 0.20, FDR p = 0.037), while precuneus/left inferior occipital gyrus (β = −5.21, SE = 1.44, r = −0.20, FDR p = 0.030) and precuneus/right middle occipital gyrus (β = −4.92, SE = 1.42, r = −0.19, FDR p = 0.039) connectivity were associated with anxiety. Furthermore, connectivity between left inferior parietal lobule and superior parietal lobule was also linked to anxiety (β = 6.49, SE = 1.69, r = 0.21, FDR p = 0.030). **c) Links between neuroticism networks and symptom severity in AURORA**. Connectivity between precuneus and left middle temporal gyrus was positively associated with stress (β = 3.89, SE = 1.47, r = 0.15, FDR p = 0.042), anxiety (β = 18.69, SE = 5.84, r = 0.18, FDR p = 0.013), and depression (β = 10.80, SE = 3.31, r = 0.18, FDR p = 0.043). Connectivity between paracentral lobule and left thalamus was also positively associated with stress (β = 18.44, SE = 5.48, r = 0.19, FDR p = 0.013) and anxiety (β = 4.16, SE = 1.35, r = 0.18, FDR p = 0.020); connectivity between right superior parietal lobule and right inferior frontal gyrus was also positively associated with stress (β = 20.26, SE = 5.93, r = 0.19, FDR p = 0.013) and anxiety (β = 4.48, SE = 1.49, r = 0.16, FDR p = 0.020). In contrast, connectivity between left inferior parietal lobule and inferior frontal gyrus connectivity was negatively associated with stress (β = −15.27, SE = 4.91, r = −0.17, FDR p = 0.013) and anxiety (β = −3.55, SE = 1.21, r = −0.16, FDR p = 0.003). Connectivity between precuneus and right middle occipital gyrus was negatively related to stress (β = −18.61, SE = 5.76, r = −0.18, FDR p = 0.013) and anxiety (β = −4.92, SE = 1.42, r = −0.19, FDR p = 0.011), and connectivity between precuneus and left inferior occipital gyrus was also negatively related to stress (β = −17.99, SE = 5.84, r = −0.17, FDR p = 0.013) and anxiety (β = −5.21, SE = 1.44, r = −0.20, FDR p = 0.011). Finally, connectivity between precuneus and left middle temporal gyrus also showed a negative association with stress and anxiety (β = −4.83, SE = 1.54, r = −0.17, FDR p = 0.002). **d) Links between anhedonia networks and symptom severity in AURORA**. We found a positive association between left posterior cingulate cortex–left middle frontal gyrus connectivity and stress (β = 23.73, SE = 6.20, r = 0.21, FDR p = 0.043), and a negative association between left putamen–left middle frontal gyrus connectivity and stress (β = −21.70, SE = 6.00, r = −0.20, FDR p = 0.048). Precuneus–left insula connectivity was positively linked to depression (β = 15.38, SE = 3.85, r = 0.22, FDR p = 0.022). No significant associations with anxiety were found for anhedonia-related networks.

We used a GLM to examine associations between the brain networks linked with cognitive function in UKBB and symptom severity related to stress, anxiety, and depression assessed two weeks after trauma exposure in individuals from the AURORA study (**Supplementary Fig. 5**). The model included age, sex, age^2^, age × sex interaction, income, years of education, site, and type of trauma as covariates. Figure 3a illustrates the reaction time networks alongside measures of stress, anxiety, and depression (**Supplementary Data 5, 6, and 7**). Among the 28 connectivity measures related to reaction time in UKBB, only connectivity between left posterior cingulate cortex and left middle frontal gyrus was significantly associated with stress measures (i.e., PCL-5) in AURORA after FDR correction (*β* = 23.73, *SE* = 6.20, *r* = 0.21, *FDR p* = 0.0047). No significant associations were found between these brain networks and measures of anxiety or depression after FDR correction.

Figure 3b depicts the relationship between the numeric memory network identified in UKBB and symptom severity in AURORA (**Supplementary Data 8, 9, and 10**). Our results indicate that connectivity between left posterior cingulate cortex and left anterior cingulate cortex was significantly associated with stress measured with PCL-5 (*β* = 24.03, *SE* = 5.54, *r* = 0.20, *FDR p* = 3e^− 4^), anxiety measured with PROMIS anxiety (*β* = 5.00, *SE* = 1.37, *r* = 0.20, *FDR p* = 0.030), and depression measured with PROMIS depression (*β* = 11.81, *SE* = 3.08, *r* = 0.21, *FDR p* = 0.038) in AURORA participants. Additionally, connectivity between right inferior parietal lobe and right inferior frontal gyrus was associated with stress (*β* = 23.18, *SE* = 6.33, *r* = 0.20, *FDR p* = 0.037), while precuneus and left inferior occipital gyrus connectivity and precuneus and right middle occipital gyrus connectivity were significantly associated with anxiety (precuneus/inferior occipital gyrus: *β* = −5.21, *SE* = 1.44, *r* = −0.20, *FDR p* = 0.030; precuneus/right middle occipital gyrus: *β* = −4.92, *SE* = 1.42, *r* = −0.19, *FDR p* = 0.039). Furthermore, connectivity between left inferior parietal lobule and superior parietal lobule was significantly associated with anxiety measures (*β* = 6.49, *SE* = 1.69, *r* = 0.21, *FDR p* = 0.030).

### Emotion networks link with symptom severity in trauma-exposed individuals

Next, we developed GLMs to examine associations between brain networks associated with emotion dysregulation (i.e., neuroticism and anhedonia) in the UKBB and the severity of stress, anxiety, and depression symptoms in AURORA. Figure 3c illustrates links between the neuroticism network and stress, anxiety, and depression (**Supplementary Data 11, 12, and 13**). We found positive associations between precuneus/left middle temporal gyrus connectivity and stress (*β* = 3.89, *SE* = 1.47, *r* = 0.15, *FDR p* = 0.042), anxiety (*β* = 18.69, *SE* = 5.84, *r* = 0.18, *FDR p* = 0.013), and depression (*β* = 10.80, *SE* = 3.31, *r* = 0.18, *FDR p* = 0.043). Significant associations were also observed between paracentral lobule/left thalamus connectivity and stress (*β* = 18.44, *SE* = 5.48, *r* = 0.19, *FDR p* = 0.013) and anxiety (*β* = 4.16, *SE* = 1.35, *r* = 0.18, *FDR p* = 0.020). Similarly, right superior parietal lobule/right inferior frontal gyrus connectivity significantly linked with stress (*β* = 20.26, *SE* = 5.93, *r* = 0.19, *FDR p* = 0.013) and anxiety (*β* = 4.48, *SE* = 1.49, *r* = 0.16, *FDR p* = 0.020). Conversely, left inferior parietal lobule/inferior frontal gyrus connectivity showed a significant negative link with stress (*β* = −15.27, *SE* = 4.91, *r* = −0.17, FDR p = 0.013) and anxiety (*β* = −3.55, *SE* = 1.21, *r* = −0.16, *FDR p* = 0.003). Negative links were also found between precuneus/right middle occipital gyrus and stress (*β* = −18.61, *SE* = 5.76, *r* = −0.18, *FDR p* = 0.013) and anxiety (*β* = −4.92, *SE* = 1.42, *r* = −0.19, *FDR p* = 0.011), as well as between precuneus/left inferior occipital gyrus and both stress (*β* = −17.99, *SE* = 5.84, *r* = −0.17, *FDR p* = 0.013) and anxiety (*β* = −5.21, *SE* = 1.44, *r* = −0.20, *FDR p* = 0.011). Finally, a negative link was identified between precuneus/left middle temporal gyrus and both stress and anxiety (*β* = −4.83, *SE* = 1.54, *r* = −0.17, *FDR p* = 0.002).

Figure 3d shows links between anhedonia networks identified in the UKBB and stress, anxiety, and depression measures in AURORA (**Supplementary Data 14, 15, and 16**). We found a positive association between left posterior cingulate cortex/left middle frontal gyrus connectivity and stress (*β* = 23.73, *SE* = 6.20, *r* = 0.21, *FDR p* = 0.043) and a negative association between left putamen/left middle frontal gyrus connectivity and stress (*β* = −21.70, *SE* = 6.00, *r* = −0.20, *FDR p* = 0.048). Additionally, a positive association was observed between precuneus/left insula activity and depression (*β* = 15.38, *SE* = 3.85, *r* = 0.22, *FDR p* = 0.022). After FDR correction, no significant associations emerged between anhedonia networks and anxiety measures in the AURORA sample.

#### Emotion and cognition network dynamics linked with symptom severity in trauma-exposed individuals

**Figure 4:**
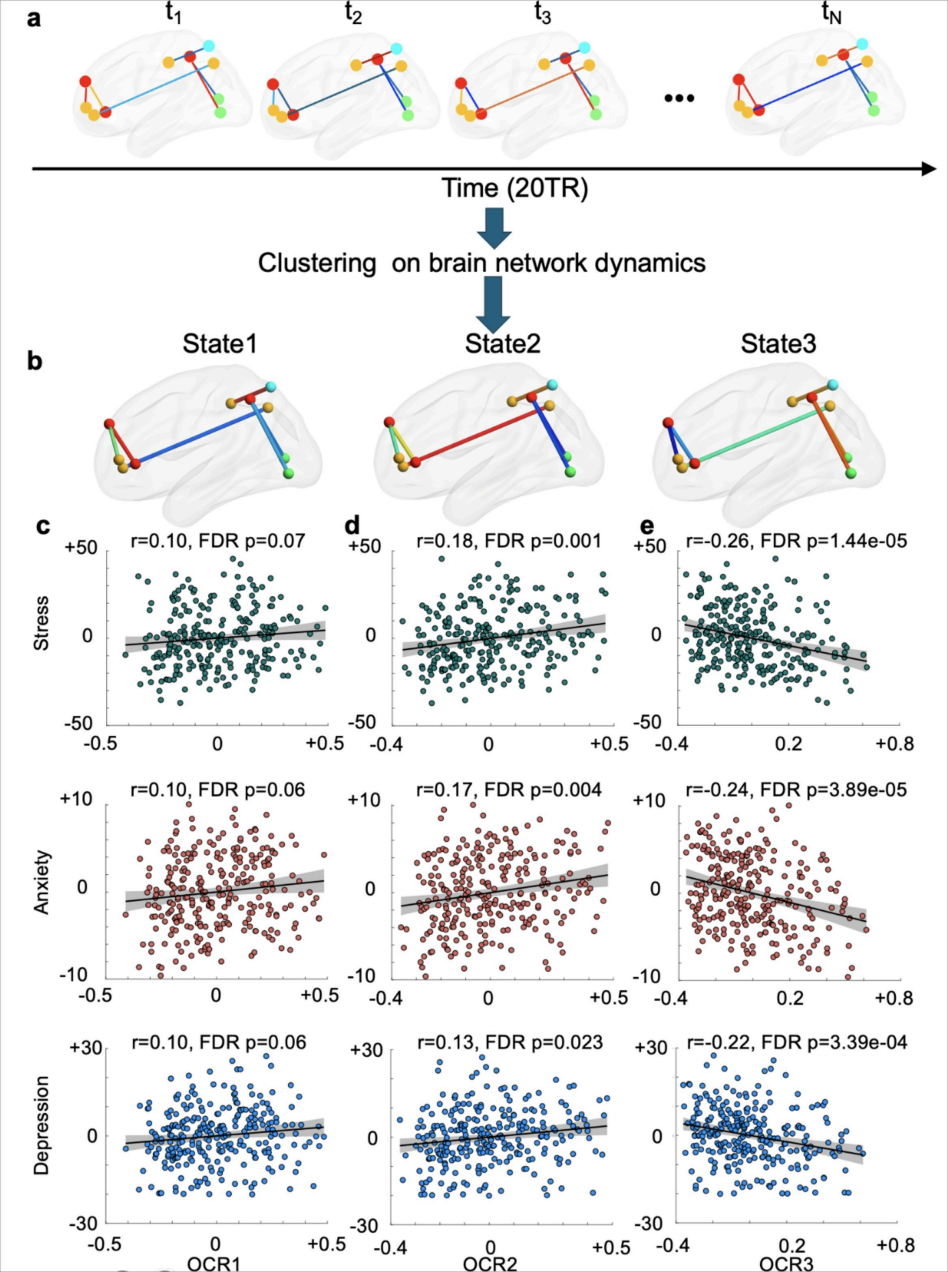
Temporal dynamic functional connectivity analyses of cognition network. **a)** Dynamics of the cognitive network identified by analyzing UKBB and AURORA data. **b)** After calculating all dynamic functional connectivity measures for AURORA participants, we aggregated them into three clusters or states. For each participant, a state vector was determined, indicating the network’s state at any given time. We also assessed the occupancy rate (or OCR), which indicates the proportion of time each participant spent in each state, resulting in three OCRs per participant. Finally, we employed GLMs to explore relationships between these OCRs and symptom severity (i.e., stress, anxiety, and depression). Links between stress, anxiety, and depression measures in AURORA and **c)** OCR in state 1, **d)** OCR in state 2, and **e)** OCR in state 3. Spending more time in this state characterized by strong connectivity between precuneus and visual network regions was linked to lower symptom severity.

**Figure 5:**
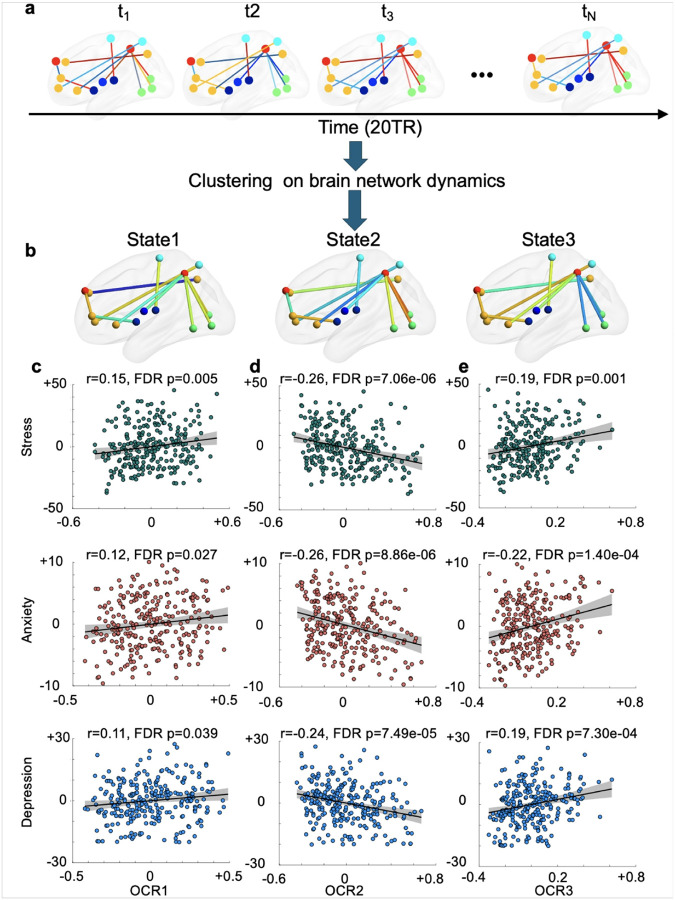
Temporal dynamic functional connectivity analyses of the emotion network. **a)** Dynamics of the emotion network identified by analyzing UKBB and AURORA data. **b)** After calculating dynamic functional connectivity measures for the AURORA participants, we aggregated them into three clusters or states. For each participant, a state vector was determined, indicating the network’s state at any given time point. Furthermore, we assessed the occupancy rate (OCR), which indicates the proportion of time each participant spent in each state, resulting in three occupancy rates for each participant. Finally, we employed GLMs to explore the relationship between these OCRs and symptom severity (i.e., stress, anxiety, and depression). Links between stress, anxiety, and depression measures in AURORA and **c)** OCR in state 1 **d)** OCR in state 2, and **e)** OCR in state 3. Spending more time in this state characterized by weaker connectivity between the precuneus and visual network regions was linked to higher symptom severity.

We next examined whether the temporal dynamics of the cognitive and emotional networks identified in the UKBB cohort were associated with symptom severity in AURORA. To analyze network dynamics, we used a previously developed pipeline^66–70^. Initially, we segmented neural time series data using a sliding window approach^71,72^. We then calculated functional connectivity within each window for the cognitive and emotion networks, as illustrated in Fig. 4a (for cognition) and Fig. 5a (for emotion). For each AURORA participant, we analyzed 210 windows. After calculating all dynamic functional connectivity measures we aggregated them into three clusters, called ‘states’ in the dynamic functional connectivity literature (**Supplementary Fig. 8**). For each participant, a state vector was determined, indicating the network’s state at any given time. From each individual state vector, we assessed the occupancy rate (OCR), which indicates the proportion of time each participant spent in each dynamic functional connectivity state, resulting in three OCRs (called OCR1 for state 1, OCR2 for state 2, and OCR3 for state 3) per participant. Finally, we employed GLMs to explore relationships between OCRs and symptom severity (i.e., stress, anxiety, and depression), again including age, sex, age^2^, age × sex, income, years of education, site, and type of trauma as covariates.

Figure 4b illustrates the three *cognitive* states identified in the AURORA dataset. In this figure, the color bar represents the strength of connectivity, where warmer colors indicate positive connectivity and cooler colors signify negative connectivity. Figure 4c shows relationships between the occupancy rate of state 1 (OCR1) and symptoms of stress (top), anxiety (middle), and depression (bottom) in AURORA participants. Although we observed a positive correlation between OCR1 and symptom severities, none remained significant after FDR correction We did, however, observe significant positive associations between OCR2 and stress (*β* = 18.39, *SE* = 5.49, *r* = 0.13, *FDR p* = 0.001), anxiety (*β* = 4.11, *SE* = 1.135, *r* = 0.17, *FDR p* = 0.004), and depression (*β* = 7.73, *SE* = 3.16, *r* = 0.13, *FDR p* = 0.023), as shown in Fig. 4d. This indicates that participants who spent more time in state 2 had greater symptom severity related to stress, anxiety, and depression. By contrast, Fig. 4e shows a negative correlation between OCR3 and measures of stress (*β* = −22.24, *SE* = 4.74, *r* = −0.26, *FDR p* = 1.44e^− 5^), anxiety (*β* = −5.27, *SE* = 1.18, *r* = −0.24, *FDR p* = 3.89e^− 5^), and depression (*β* = −10.84, *SE* = 2.76, *r* = −0.22, *FDR p* = 3.39e^− 4^). This indicates that participants spending more time in this state show reduced symptom severity.

Figure 5b displays the three *emotion* states identified in the AURORA dataset. A positive relationship was observed between the occupancy rate of state 1 (OCR1) and measures of stress (*β* = 14.71, *SE* = 5.26, *r* = 0.15, *FDR p* = 0.005), anxiety (*β* = 2.91, *SE* = 1.31, *r* = 0.12, *FDR p* = 0.027), and depression (*β* = 6.22, *SE* = 3.01, *r* = 0.11, *FDR p* = 0.039), as shown in Fig. 5c. This indicates that participants spending more time in state 1 exhibited higher symptom severity. Conversely, a negative correlation emerged between OCR2 and stress (*β* = −19.75, *SE* = 4.07, *r* = −0.26, *FDR p* = 7.06e^− 6^), anxiety (*β* = −4.90, *SE* = 1.02, *r* = −0.26, *FDR p* = 8.86e^− 6^), and depression (*β* = −10.21, *SE* = 2.37, *r* = −0.24, *FDR p* = 7.49e^− 5^) measures, as illustrated in Fig. 5d. This indicates that participants spending more time in state 2, characterized by greater connectivity between precuneus and regions from VIS, tended to show lower symptom severity.

Additionally, a positive relationship was identified between OCR3 and stress (*β* = 20.06, *SE* = 5.82, *r* = 0.19, *FDR p* = 0.001), anxiety (*β* = 5.69, *SE* = 1.43, *r* = 0.22, *FDR p* = 1.40e^− 4^), and depression (*β* = 11.80, *SE* = 3.34, *r* = 0.19, *FDR p* = 7.30e^− 4^) measures, as demonstrated in Fig. 5e. This implies that participants spending more time in state 3, characterized by weaker connectivity between precuneus and regions from VIS, experienced higher symptom severity.

## Discussion

This study introduces a scalable, interpretable route from population functional connectivity to early post-trauma symptomatology. We first derived functional connectivity profiles tied to cognition (reaction time; numeric memory) and emotion (neuroticism; anhedonia) in > 14,000 healthy adults, then projected an independent, clinically heterogeneous AURORA cohort into this reference to determine whether and how the connectivity profiles related to symptoms expressed two weeks post-trauma. Across analyses, the precuneus—a high-degree hub of the DM network with VIS and CC interfaces—emerged as critical. Stronger precuneus–VIS coupling and greater occupancy of a “precuneus-engaged” dynamic state tracked lower stress, anxiety, and depression, whereas weaker coupling and greater occupancy of a “precuneus-weak” state tracked higher burden. These findings indicate that failures of cognition and emotion regulation in the early aftermath of trauma reflect disrupted coordination among DM, VIS, and CC systems, rather than dysfunction localized to a single circuit.

To relate brain connectomes to behavior, prior studies have typically employed either hypothesis-driven analyses that focus on *a priori* circuits for greater interpretability (but low discovery)^52,53^ or fully datadriven approaches that enable broad discovery but offer limited mechanistic insight^54,55^. Both approaches have strengths but also weaknesses, including multiple-comparison burden and interpretability gaps with a purely data-driven approach^73^. Our hybrid *discover-then-project* strategy combines the strengths of both: we first discover functional connectivity and behavior associations in a large, healthy reference cohort, then constrain tests in trauma-exposed individuals to those circuits, anchoring the tests to key circuits—some newly discovered—while retaining sensitivity to unanticipated relationships. Although used with a trauma-exposed sample here, the framework is general and applicable to other populations and questions.

In the UKBB, reaction time related to coupling within and between CC, VIS, and DM, with CC (frontal–parietal) contributions most prominent. Faster responses were linked to stronger within-CC and CC–DM/VIS coupling, and to increased VIS and VIS–SM connectivity, consistent with efficient sensory encoding and sensorimotor coordination. Follow-up work should probe causal mechanisms and error-driven control (e.g., post-error adjustments)^71,72^. Numeric memory implicated 243 connections, 43% within CC, consistent with a key role for fronto-parietal circuits in processing capacity and efficiency^74–77^. Also, VIS coupling predicted better performance, aligning with models in which occipito-parietal pathways support rapid encoding and interference control^78^. DM and SM nodes also contributed, indicating that numeric memory depends on cross-network coordination between internally oriented processes (DM)^79^, self-referential evaluation^80^, and rapid sensorimotor processing^81^. A contrasting pattern emerged for the cerebellar (CB) network. Although the cerebellum is traditionally linked to motor control, growing evidence highlights its role in cognition^82–84^. In this context, reduced cerebellar connectivity may indicate more streamlined neural processing^85–87^, potentially by fine-tuning or filtering out irrelevant signals^88^. This suggests that, under certain conditions, lower cerebellar integration may enhance cognitive efficiency. Whether this marks task specificity or a broader principle warrants direct testing.

Emotion-related organization showed complementary structure. DM and CC networks emerged as key contributors, each showing distinct patterns linked to neuroticism. In the CC, connectivity varied with neuroticism, with some connections increasing and others decreasing, and CC coupling with VIS and SM also strengthened. This contrasts with a prior study of 120 women that found minimal involvement of sensorimotor and CC subnetworks^89^, highlighting broader network engagement in our larger sample. Higher neuroticism was also associated with reduced integration between the DM and other key networks, including CC, SM, VIS, and AUD. These reductions suggest disrupted coordination across brain network essential for emotion regulation^90^. The impaired connectivity may affect the processing of self-referential thought, cognitive control, sensory input, or attention, which are all critical for emotional stability. The precuneus, a central DM node, showed the strongest negative shifts in connectivity, indicating its potential as a neural marker of neuroticism-related dysfunction. Prior research supports this, reporting reduced precuneus connectivity^91^ and activity^92^ in individuals with higher neuroticism. Overall, higher neuroticism is characterized by diminished DM integration, particularly involving the precuneus, and increased CC coupling with sensory networks, reflecting distinct patterns underlying emotion regulation difficulties. Moreover, we found that anhedonia involves widespread changes in brain connectivity. Some networks showed increased integration, particularly subcortical regions like the caudate, hypothalamus, putamen, and thalamus, suggestive of over-engagement. In contrast, the CB showed reduced connectivity, mirroring patterns seen in neuroticism. These findings point to a reorganization of reward and emotion regulation circuits in anhedonia.

In the AURORA dataset, we asked whether UKBB-derived circuits for cognition and emotion are relevant to early post-trauma symptoms. After covariate adjustment and FDR correction, a focused subset met significance (1/28 RT edges, 5/239 numeric-memory edges, 7/34 neuroticism edges, and 3/262 anhedonia edges), indicating selective, directionally mixed associations between brain connectivity and symptoms rather than a uniform gain or loss of connectivity. Two complementary motifs organize the pattern. First, *hyperintegration* was observed at DM–control/limbic interfaces, reflected in stronger coupling of posterior cingulate cortex–middle frontal gyrus, posterior cingulate cortex–anterior cingulate cortex, inferior parietal lobule–inferior frontal gyrus, superior parietal lobule–right inferior parietal lobule, precuneus–middle temporal gyrus, and precuneus–insula, tracked higher symptom burden, possibly related to strong associations between self-referential thought and control/affective systems that may amplify perseverative negative thought and yield inefficient allocation of control. Second, *hypointegration* was observed at DM–visual interfaces, reflected in weaker precuneus–middle occipital gyrus, precuneus–inferior occipital gyrus, precuneus–middle temporal gyrus, and middle frontal gyrus–putamen, also tracked higher burden, suggesting a loss of sensory tethering that normally constrains internal narratives. Notably, the precuneus–middle temporal gyrus edge appeared with opposite signs across the discovery axes, implying that symptom expression can arise via distinct routes: excessive coupling along a semantic/self-referential channel versus insufficient coupling along a perceptual/episodic channel. Together, these motifs point to a biphasic failure of coordination centered on the precuneus, with too much coupling to control/limbic partners and too little coupling to visual pathways, an interpretation that aligns with prior demonstrations of large-scale connectivity disruption in stress^93,94^, anxiety^95,96^, and depression^97^ while specifying where (DM–VIS and DM/CC interfaces) and how (overbinding versus loss of visual constraint) coupling goes awry.

Across analyses, the precuneus emerged as the place where cognition- and emotion-related circuitry intersected, not simply as a highly connected node but as an area where internally generated thought can be calibrated against sensory evidence. In static functional connectivity, the precuneus sat at the interface of DM with VIS; in the dynamic analysis, time spent in a precuneus–VIS engaged state aligned with lower symptoms, whereas time in a precuneus-weak state aligned with higher burden. This pattern suggests a mechanistic role in gating: when precuneus-centered coupling to VIS is strong, visual context constrains self-referential and mnemonic processing, supporting flexible shifts between internal goals and external demands; when that coupling weakens, internal narratives are less tethered to sensory input, enabling rumination, threat-biased appraisal, and inefficient control. This account is consistent with evidence that the precuneus contributes to emotional working memory^90^ and, as a core DM node, integrates sensory inputs with abstract association areas to support both internally and externally directed cognition^98,99^. It also aligns with recent reports that implicate the precuneus in emotional regulation, memory, and sensory integration^100,101^.

These features suggest a translational pathway. The precuneus is a hub where strengthening DM–VIS coupling could restore the balance between internal models and sensory constraints. Stimulation studies in other populations indicate that precuneus-targeted repetitive transcranial magnetic stimulation or rTMS can enhance cognition^102–104^, and our dynamic results specify a direction of change to pursue: shift occupancy away from a precuneus-weak configuration toward a precuneus–VIS engaged configuration. This can be operationalized as a target-engagement strategy, with state occupancy as a proximal biomarker and with complementary behavioral approaches, such as working memory training, to bias the system toward adaptive coupling patterns^105^. Together, these insights position precuneus-centered integration as both a mechanism and a modifiable intermediate phenotype at the intersection of stress-related cognitive and emotional symptoms.

A key limitation of this study is the incomplete consideration of environmental variables in the UKBB modeling. While we adjusted for core demographic factors, we did not include other relevant environmental factors, such as socioeconomic status, early life adversity, and lifestyle habits, that may influence brain connectivity and cognitive-emotional processes. Additionally, the age range differs between the UKBB and AURORA cohorts, which may impact the generalizability of our findings despite our efforts to adjust for age and age^2^ in all models. Furthermore, due to data availability, we included only a limited set of cognitive measures and emotion-related proxies, which do not fully capture the complexity of cognitive and emotional functioning. Future studies should incorporate a broader range of cognitive and emotional measures and include more comprehensive environmental factors to better understand the robustness and applicability of these results. Finally, the UKBB rs-fMRI data were collected for approximately 6 minutes. While some studies suggest 5–7 minutes of rs-fMRI data can provide stable functional connectivity measurements^106^, others recommend extending the scan length to 9–13 minutes or more to improve test-retest reliability^107^.

Future work should: (1) integrate task probes of controllability, set-shifting, and explicit emotion regulation to link resting dynamics to performance and causal models of network controllability; (2) combine functional connectivity with structural connectivity, microstructure, and physiological measures (e.g., HRV, pupillometry) to model body–brain loops that shape DM–VIS–CC coupling; and (3) conduct target-engagement studies that manipulate precuneus-centered coupling (using rTMS, real-time fMRI neurofeedback, or working-memory/attentional-control training), quantify shifts in state occupancy as proximal endpoints, and evaluate symptom change. Together, these steps could operationalize a circuitfirst pathway from population mapping to personalized care, to test whether restoring DM–VIS–precuneus coordination ameliorates cognition–emotion failures across stress-related psychopathology.

## Methods

### Inclusion and ethics statement

Ethical approval for the UK Biobank was originally granted by the Northwest Centre for Research Ethics Committee (reference 11/NW/0382). The present study was conducted under UK Biobank application number 34175. The AURORA study was conducted in accordance with ethical guidelines and received approval from the Institutional Review Board (IRB) at the University of North Carolina (IRB no. 1707–03) on 12 May 2017, covering multiple sites. Additional sites either entered into reliance agreements or conducted parallel IRB reviews. Participants provided written informed consent prior to participation. An independent medical monitor evaluated and approved the procedures for handling any cases of clinical deterioration reported by participants or identified by study staff. This monitor also reviewed detailed reports of participant interactions prepared by experienced clinicians.

### Study population

Our study includes two datasets from UKBB and AURORA. We used UKBB as our reference healthy cohort to identify brain networks associated with cognition measures such as choice reaction time and numeric memory, and emotion dysregulation measures such as neuroticism and anhedonia. In the UKBB, we focused on individuals aged 45–85 years old who met MRI imaging quality standards. We excluded individuals with the following primary or secondary diagnosis: delirium, not induced by alcohol and other psychoactive substances (F05); other mental disorders due to brain damage and dysfunction and to physical disease (F06); personality and behavioral disorders due to brain disease, damage and dysfunction (F07); unspecified organic or symptomatic mental disorder (F09); mental and behavioral disorders due to psychoactive substance use (F10-F19); schizophrenia, schizotypal and delusional disorders (F20–29); manic episodes (F30); or bipolar affective disorder (F31). These diagnoses are coded according to the International Classification of Diseases version 10 (ICD-10). We also excluded individuals with specified diagnoses and those who had sought general practitioner or psychiatric consultation for “nerves, anxiety, tension, or depression”. See the Supplementary Information for more detail about this process. After these exclusions, our cohort includes 14,047 participants, comprising 6,362 males and 7,685 females with a mean age of 64.22 ± 7.54 years. We used this cohort to identify brain networks associated with our measures of interest in the healthy population (see **Supplementary Fig. 1**).

Additional data came from adults who were recruited as part of the multi-site Emergency Department (ED) AURORA study^62^. The study targeted individuals who had experienced a traumatic event necessitating an ED evaluation, with recruitment occurring within 72 hours of the event^62^. This cohort of early post-trauma participants was selected to explore changes in brain function that could heighten the risk for trauma-related psychopathology in the subsequent weeks or months. The study aimed to enroll a demographically representative sample of the US population without restrictions on sex, gender, race, or ethnicity. In the AURORA study, participants who experienced incidents like a car accident, a high fall (> 10 feet), a physical assault, sexual violence, or a mass casualty incident were considered as having experienced trauma. The inclusion criteria included: 1) between 18 and 75 years old; 2) being alert and oriented at the ED; 3) able to speak and write English fluently; 4) no cognitive impairment; and 5) ability to use a smartphone for > 1-year post-enrollment. Exclusion criteria included solid organ damage, severe bleeding, requirement for a chest tube, and the likelihood of being admitted for longer than 72 hours. The study eventually included 2,943 AURORA participants with clinical item-level data, recruited between September 2017 and June 2021, marking the final data freeze for psychometric release (Freeze 4.0 dataset at September 22nd, 2021). Participants were recruited at participating ED locations and directed to one of five ‘deep phenotyping’ sites, where they were invited to undergo MRI scans. Scans were conducted in the morning or afternoon, approximately two weeks following the traumatic event (i.e., WK2). After thorough preprocessing and quality checks, data from 306 participants were included in our study (see **Supplementary Fig. 2**).

In the AURORA dataset, the PTSD Checklist for DSM-5 (PCL-5)^64^ was administered to assess PTSD symptoms at multiple time points: week 2 (WK2), week 8 (WK8), month 3 (M3), month 6 (M6), and month 12 (M12). Anxiety levels were evaluated using the Patient-Reported Outcomes Measurement Information System (PROMIS) Anxiety Bank Items^65^ at all these time points. PROMIS is a set of tools that evaluates physical, mental, and social health, providing a standardized approach to measure patient outcomes effectively. Depression was assessed at each time point using the PROMIS Depression scale^65^. The current study specifically focuses on the WK2 data, which aligns with the timing of imaging data acquisition. The distribution of PCL-5, PROMIS anxiety, and PROMIS depression is shown in **Supplementary Fig. 5**.

### Measures of cognitive function in UKBB

In the current study we focused on two measures of cognitive function. The touch-screen test of reaction time (“Snap” test), administered at the UK Biobank Assessment Centers, is designed to measure participants’ choice reaction time. During the test, participants view pairs of cards on a touchscreen and must press a button as quickly as possible when the cards are identical. This test includes 12 rounds, with each round presenting a pair of cards. Here we focus on mean reaction time on correct trials; we selected this measure because reaction time, especially in paradigms requiring a choice, is consistently correlated with measures of intelligence^108^, which is broadly indicative of general cognitive ability. **Supplementary Fig. 4a** shows the distribution of mean reaction time in the UKBB dataset.

As a second measure of cognitive function, we selected the UK Biobank’s Numeric Memory test, which measures numeric memory, or more precisely, immediate recall of increasingly longer strings of numbers while under time constraint. During the test, the participant is shown a 2-digit number, which is displayed for 2000 ms plus (the number of digits [i.e. 2] × 500 ms). During this time, the on-screen keyboard is inactive. The number then disappears and, after a wait period of 3000 ms, the participant is asked to enter the remembered number using a keyboard. Once a number has been entered and the participant has touched the “Next” button, the entered number is removed, and the keyboard is deactivated for 600ms before either displaying the next number or ending the test. If the participant recalls the number correctly, the next number shown increases by one digit, incrementally increasing the difficulty. The test continues in this manner and can progress up to a 12-digit number. The Numeric Memory test thus measures working memory capacity and also assesses the ability to focus and perform under time constraints, as each number is displayed for a limited time. The data collected includes the maximum length of the number, response accuracy, and total duration of the test. **Supplementary Fig. 4b** shows the distribution of numeric memory scores in the UKBB dataset.

### Emotion regulation measures in UKBB

In the UK Biobank study, neuroticism—a personality trait associated with emotional instability and a propensity to experience negative feelings—was assessed using a touch-screen questionnaire. Participants viewed a series of questions aimed at identifying characteristics commonly associated with neuroticism, such as mood fluctuations, irritability, and susceptibility to stress. The questions included prompts like “Do you ever feel ‘just miserable’ for no reason?” and “Are you often troubled by feelings of guilt?” Participants could respond with “Yes,” “No,” “Do not know,” or “Prefer not to answer”. Neuroticism was quantified by summing the number of “Yes” responses across the twelve questions, yielding a total score for each participant. **Supplementary Fig. 4c** shows the distribution of neuroticism scores. Additionally, we identified 4,379 participants with anhedonia in the UK Biobank. The inclusion criteria included participants who had either seen a general practitioner (or a psychiatrist) for nerves, anxiety, tension, or depression, and those who reported feeling unenthusiastic or disinterested for two weeks or longer (see *Supplementary Materials*). We chose this approach because no dimensional measure of anhedonia is available in the UK Biobank. Note while we refer to these as measures of emotion regulation, in fact they measure *dys*regulation of negative (neuroticism) and positive (anhedonia) emotions; consequently, lower scores can be interpreted as reflecting better emotion regulation.

### Imaging acquisition protocol

The UKBB imaging data were collected on the same day as the assessments for cognition and emotion using Siemens Skyra 3T scanners at three different centers. To ensure data compatibility, identical scanners and protocols were used across the centers. Each rs-fMRI session lasted approximately 6 minutes, with participants instructed to focus on a fixation cross to minimize eye movements and promote consistent data collection. Detailed imaging protocols, designed by the UK Biobank Imaging Working Group, can be found in https://biobank.ctsu.ox.ac.uk/crystal/crystal/docs/brain_mri.pdf.

For the AURORA study, participants underwent comprehensive screening to rule out any contraindications to MRI, among the other exclusion criteria described earlier. Individuals who could potentially be pregnant were given a pregnancy test before entering the MRI environment. Although the scan sequences were consistent across sites, some variations in sequence parameters occurred due to hardware differences; The imaging protocol for each site involved in AURORA and UKBB is detailed in **Supplementary Table 4**. The resting-state imaging procedure lasted about 9 minutes, with participants asked to keep their eyes open, focus on a fixation cross, and remain still^109^.

### Preprocessing

We corrected for differences in image acquisition times between slices using the default slice timing routines in Statistical Parametric Mapping (SPM12 @ https://www.fil.ion.ucl.ac.uk/spm/). The slice acquired in the middle of the sequence was designated as the reference slice. Head motion was then corrected using a rigid body transformation, with mean square difference as the cost function, and 3D brain translations and rotations were estimated. Subsequently, the imaging data were resampled to a 3 × 3 × 3 mm^³^ resolution and spatially normalized to Montreal Neurological Institute (MNI) space using the MNI152 template with the echo-planar imaging (EPI) template and the default bounding box from the SPM toolbox. The fMRI images were smoothed with a Gaussian kernel of 6 mm full width at half maximum (FWHM). A similar preprocessing strategy has been used in several of our previous studies^69,70,110–112^.

### Extracting independent components using NeuroMark

To obtain reliable independent components (ICs), we used the fully automated NeuroMark ICA pipeline, which incorporates previously derived component maps as priors for spatially constrained ICA. The NeuroMark framework is based on the NeuroMark template, which was constructed from two large datasets: the Human Connectome Project (HCP: https://www.humanconnectome.org/study/hcp-young-adult/document/1200-subjects-data-release, 823 subjects after selection) and the Genomics Superstruct Project (GSP: https://dataverse.harvard.edu/dataverse/GSP, 1005 subjects after selection). This framework has been successfully applied to a variety of studies, identifying brain imaging markers across different brain conditions^69,70,110–112^. The construction of the templates is detailed in our previous NeuroMark publication^113^. The NeuroMark template includes 53 independent components (ICs), which are organized into seven functional networks based on anatomical and functional knowledge (Fig. 1c). These networks include the subcortical network (SC), auditory network (AUD), sensorimotor network (SM), visual network (VIS), cognitive control network (CC), default-mode network (DM), and cerebellar network (CB). All 53 ICs and their coordinates are listed in **Supplementary Table 3**. We used these priors (i.e., the Neuromark_fMRI_1.0 template, available in GIFT @ http://trendscenter.org/software/gift and on the TReNDS website @ http://trendscenter.org/data) to perform a fully automated ICA analysis in GIFT v4.0.5.14^114^. Additionally, we: 1) detrended linear, quadratic, and cubic trends; 2) performed multiple regression on the six realignment parameters and their temporal derivatives; 3) de-spiked detected outliers; and 4) applied a low-pass filter (with a cutoff frequency of 0.15 Hz) to eliminate noise and artifacts.

### Functional connectivity estimation

To estimate functional network connectivity (FNC), we calculated the Pearson correlation between pairs of ICs in each subject as shown in Eq. 1

1 $$R=\frac{\sum_{n=1}^{N}\left(x_{1}-\bar{x_{1}}\right)\left(x_{2}-\bar{x_{2}}\right)}{\sqrt{\sum_{n=1}^{N}{\left(x_{1}-\bar{x_{1}}\right)}^{2}}\sqrt{\sum_{n=1}^{N}{\left(x_{2}-\bar{x_{2}}\right)}^{2}}}$$

where *x*1 and *x*2 are time course signals and $\bar{x_{1}}$ and $\bar{x_{2}}$ are the means of *x*1 and *x*2, respectively. This equation takes values in the interval [− 1, 1] as measures of the strength of the linear relationship between *x*1 and *x*2. Each FNC is a 53×53 matrix, from which we derived a total of 532=1378 connectivity features.

### Dynamic functional connectivity analysis

The dynamic functional connectivity of cognition and emotion networks was estimated using a sliding window approach, as illustrated in **Supplementary Fig. 7**. We employed a tapered window created by convolving a rectangular window (size = 20 TRs = 47.2 s) with a Gaussian (σ = 3) for the dynamic functional connectivity measure. Prior research suggests a window size between 30 and 60 seconds is effective for capturing variations in dynamic functional connectivity^115^. Therefore, we chose a window size of 47.2 seconds as appropriate. Within each window, Pearson correlation was used to assess functional connectivity among all regions of interest. For each participant, we computed dynamic functional connectivity for 210 windows. We aggregated the dynamic functional connectivity data across all participants and applied k-means clustering algorithm to the dynamic functional connectivity matrices organizing them into distinct clusters representing transient connectivity “states” (as shown in Step 2 of **Supplementary Fig. 7**)^69,70,111,112^. To determine the optimal number of clusters, we used the elbow method, which assesses the balance between within-cluster and between-cluster distances. By testing cluster numbers from 2 to 9, we found three clusters provided the best fit^70^. Clustering was performed using Euclidean distance as the similarity measure, with 1000 iterations to ensure stability. This process identified three unique connectivity states shared across the 306 participants, along with a state vector for each individual tracking temporal fluctuations in their dynamic connectivity (see **Supplementary Fig. 8**). We then calculated the occupancy rate (OCR) for each participant, representing the proportion of time spent in each state. OCR for state *i* was computed by dividing the number of windows assigned to that state by 210 (the total number of windows), resulting in three OCR values per participant—corresponding to the three identified states.

### Modeling and statistical analysis

We used the General Linear Model (GLM) to examine how well cognitive function and emotion regulation measures related to variation in the 1,378 functional connectivity variables. We used the UKBB dataset for this analysis (Fig. 1d). Our model accounted for a variety of covariates including age, age^2^ (to capture nonlinear effects), sex, the age × sex interaction, and the site of data collection. This approach allowed us to control potential confounds might influence both functional connectivity and behavioral measures.

Upon establishing the initial predictive model, we used False Discovery Rate (FDR) correction to identify a subset of functional connectivity measures that showed statistically significant associations with the measures of cognitive function and emotion regulation. We then carried these networks forward to investigate their power to predict stress, anxiety and depression symptom severity in the AURORA dataset (Fig. 1d). This step should reveal whether specific patterns of brain connectivity related to cognitive and emotion measures in UKBB dataset generalize to symptom severity in the AURORA dataset.

### Sensitivity analysis

To assess the sensitivity of our model linking functional connectivity to cognitive function and emotion regulation, we employed bootstrapping when working with the UKBB dataset. In each of 1,000 iterations, we subsampled 70% of the data, equivalent to N = 9,833 participants. This subsampling allowed us to repeatedly evaluate associations between functional connectivity and cognitive function/emotion regulation measures across different subsamples. Following each iteration, we applied FDR correction to control for multiple comparisons. This process enabled us to identify functional connectivity variables that remained statistically significant across multiple subsamples.

## Supplementary Material

Supplementary Files

This is a list of supplementary files associated with this preprint. Click to download.

- SendietalSupMaterial2025final.docx
- SupplementaryData1.csv
- SupplementaryData2.csv
- SupplementaryData3.csv
- SupplementaryData4.csv
- SupplementaryData5.csv
- SupplementaryData6.csv
- SupplementaryData7.csv
- SupplementaryData8.csv
- SupplementaryData9.csv
- SupplementaryData10.csv
- SupplementaryData11.csv
- SupplementaryData12.csv
- SupplementaryData13.csv
- SupplementaryData14.csv
- SupplementaryData15.csv
- SupplementaryData16.csv

## Data Availability

AURORA used in this manuscript is available through the National Institute of Mental Health (NIMH) Data Archive (NDA). The NDA Collection for the AURORA Project can be found here: https://nda.nih.gov/edit_collection.html?id=2526. This content is solely the responsibility of the authors and may not reflect the official view of any of the funders or the submitters submitting original data to NDA.

## Code Availability

The code used for preprocessing, functional connectivity, and dynamic functional connectivity calculations are available at https://trendscenter.org/software/. Statistical parametric mapping (SPM 12) is available at https://www.fil.ion.ucl.ac.uk/spm/. The NeuroMark framework and the Neuromark template (Neuromark_fMRI_1.0) have been made available and incorporated into the Group ICA Toolbox (GIFT v4.0.5.14: https://trendscenter.org/software/gift/). Chord graphs are generated using the NiChord toolbox in Python (https://github.com/paulcbogdan/NiChord). The General Linear Model (GLM) code is at https://www.mathworks.com/help/stats/fitglm.html. Also, the modeling codes are available at https://github.com/msendi6/emotion_cognition_network#
